# Electrostatic Self-assembly of 0D–2D SnO_2_ Quantum Dots/Ti_3_C_2_T_*x*_ MXene Hybrids as Anode for Lithium-Ion Batteries

**DOI:** 10.1007/s40820-019-0296-7

**Published:** 2019-08-02

**Authors:** Huan Liu, Xin Zhang, Yifan Zhu, Bin Cao, Qizhen Zhu, Peng Zhang, Bin Xu, Feng Wu, Renjie Chen

**Affiliations:** 1 School of Materials Science and Engineering, Beijing Key Laboratory of Environmental Science and Engineering, Beijing Institute of Technology, Beijing, 100081 People’s Republic of China; 2 State Key Laboratory of Organic-Inorganic Composites, Beijing Key Laboratory of Electrochemical Process and Technology for Materials, Beijing University of Chemical Technology, Beijing, 100029 People’s Republic of China

**Keywords:** MXene, SnO_2_, Quantum dots, 0D–2D hybrid, Lithium-ion battery

## Abstract

MXenes, a new family of two-dimensional (2D) materials with excellent electronic conductivity and hydrophilicity, have shown distinctive advantages as a highly conductive matrix material for lithium-ion battery anodes. Herein, a facile electrostatic self-assembly of SnO_2_ quantum dots (QDs) on Ti_3_C_2_T_*x*_ MXene sheets is proposed. The as-prepared SnO_2_/MXene hybrids have a unique 0D–2D structure, in which the 0D SnO_2_ QDs (~ 4.7 nm) are uniformly distributed over 2D Ti_3_C_2_T_*x*_ MXene sheets with controllable loading amount. The SnO_2_ QDs serve as a high capacity provider and the “spacer” to prevent the MXene sheets from restacking; the highly conductive Ti_3_C_2_T_*x*_ MXene can not only provide efficient pathways for fast transport of electrons and Li ions, but also buffer the volume change of SnO_2_ during lithiation/delithiation by confining SnO_2_ QDs between the MXene nanosheets. Therefore, the 0D–2D SnO_2_ QDs/MXene hybrids deliver superior lithium storage properties with high capacity (887.4 mAh g^−1^ at 50 mA g^−1^), stable cycle performance (659.8 mAh g^−1^ at 100 mA g^−1^ after 100 cycles with a capacity retention of 91%) and excellent rate performance (364 mAh g^−1^ at 3 A g^−1^), making it a promising anode material for lithium-ion batteries.

**Electronic supplementary material:**

The online version of this article (10.1007/s40820-019-0296-7) contains supplementary material, which is available to authorized users.

## Highlights

- 0D–2D SnO_2_ quantum dots/MXene (SnO_2_ QDs/MXene) hybrids were synthesized by electrostatic self-assembly.
- MXene not only provides efficient pathways for fast transport of electrons and Li ions, but also buffers the volume change of SnO_2_ during charge/discharge process.
- The 0D–2D SnO_2_ QDs/MXene hybrids deliver high capacity, excellent cycle and rate performances as anode of lithium-ion batteries.

**Electronic supplementary material:**

The online version of this article (10.1007/s40820-019-0296-7) contains supplementary material, which is available to authorized users.

## Graphical abstract

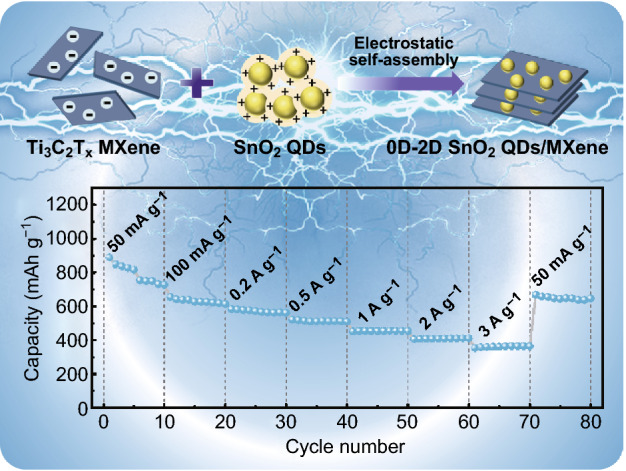

## Introduction

Lithium-ion batteries (LIBs) are widely used in various portable electronics, electric tools, and electric vehicles due to their high energy density, long cycle life, and environmental friendly [1, 2]. Nevertheless, the conventional graphite anode of LIBs with a specific capacity of 372 mAh g^−1^ can hardly meet the rapidly increasing demand of high energy density. Great efforts have been made to develop promising anode materials with high capacity, such as transition metal oxides [3, 4], alloys [5, 6], metal oxides/sulfates [7–9], and phosphorous [10–12]. Among various metal oxides, SnO_2_ has attracted a lot of attention due to its relatively high theoretical capacity (790 mAh g^−1^, which is twice than the currently used graphite), low average working potential (~ 0.6 V vs. Li^+^/Li), natural abundance and low price [13–16]. Unfortunately, its practical application as anode material in LIBs is seriously limited by the poor cycle stability resulting from the severe volume change (> 300 vol%) during the charge/discharge process. Meanwhile, SnO_2_ also suffers from low electrical conductivity, resulting in poor rate capability. To improve the electrochemical performances of SnO_2_, several strategies have been proposed to overcome these issues. One of the effective way is to fabricate nanostructured SnO_2_, such as SnO_2_ quantum dots (QDs) [17], SnO_2_ hollow spheres [18], or SnO_2_ nanowires [19], which could restrain the structure changes during lithium alloying and shorten the ion diffusion lengths [20]. The other way is to combine SnO_2_ with various carbon materials possessing high conductivity such as carbon nanotubes, carbon fiber or graphene [21–26]. The conductive carbon can not only improve the overall electrical conductivity of the composites but also act as a buffer to slow down the structure collapse of the electrode.

MXenes are a newly emerging family of 2D materials with a general formula of M_*n*+1_X_*n*_T_*x*_, where *M* represents early transition metal (*M* = Ti, Sr, V, Cr, Ta, Nb, Zr, Mo, and H_f_), X is carbon and/or nitrogen, and T stands for the surface termination groups (–OH, –F=O) [27–31]. Due to their excellent electrical conductivity, tailorable surface chemistries, and mechanical properties, MXenes have recently attracted great interests in energy storage devices such as LIBs, sodium-ion batteries, and supercapacitors [32–35]. Particularly, as one of the most widely studied MXenes, Ti_3_C_2_T_*x*_ is a potential anode material for LIBs, which can deliver excellent cycle stability and superior rate performances. However, the theoretical reversible capacity of Ti_3_C_2_T_*x*_ is only 320 mAh g^−1^ [36]. On the other hand, MXene can also be used as a substrate to fabricate hybrids with other active materials, including metal oxides, which have higher theoretical capacity but poor conductivity and are prone to large volume change during charge/discharge. In the hybrids, MXene can improve the conductivity and buffer the volume changes of the metal oxide, while the metal oxide provides high capacity. Thus, the MXene/metal oxide hybrid could achieve high capacity, stable cycle and good rate performance [37–41]. For example, Ti_3_C_2_T_*x*_/Fe_2_O_3_ nanocomposite was prepared by confining Fe_2_O_3_ nanoparticles into Ti_3_C_2_T_*x*_ nanosheets through ball-milling method, which showed reversible capacities of ~ 203 mAh g^−1^ at 1 C and 100 mAh g^−1^ at 10 C [37]. MXene/NiCo_2_O_4_ composite with a capacity reaching up to 1330 mAh g^−1^ was synthesized by spray coating method [38]. Some recent works indicate that MXene is also a good substrate to improve the lithium storage performance of SnO_2_. The hybrids of SnO_2_ with MXene prepared by wet hydrothermal approach has shown a high capacity of 1021 mAh g^−1^ and improved cycle stability [40]. However, a few reports indicate that MXene is unavoidable to be oxidized to titanium dioxide (TiO_2_) as a by-product during these processes, thereby, influencing the electrochemical performance of the composites. Recently, our group proposed a general route to self-assemble transition metal oxide nanostructures on Ti_3_C_2_T_*x*_ MXene nanosheets through van der Waals interaction. The proposed method allowed fabrication of hybrids without getting MXene to be oxidized and achieved enhanced cycle and rate performance [15].

In this work, a simple method is proposed to prepare 0D–2D SnO_2_ QDs/MXene hybrids by electrostatic self-assembling SnO_2_ QDs on the surface of 2D Ti_3_C_2_T_*x*_ MXene nanosheets under ultrasonication. Since, the synthesis is accomplished in a very mild condition, i.e., ultrasonication treatment of the mixture of negatively charged MXene and positively charged SnO_2_ QDs at room temperature, the MXene oxidation is effectively avoided. In the hybrids, 2D nanosheet of MXene acts as a substrate to support 0D SnO_2_ QDs, which can not only provide large electrode/electrolyte interface area for fast reversible transport of electrons and ions, but could also inhibit the aggregation of SnO_2_ QDs and buffer the volume changes during charge/discharge process. The SnO_2_ QDs with ultra-small particle size can effectively maximize the activity and specific capacity and minimize the volume change while inhibiting the structure collapse during charge/discharge process and shortening the lithium diffusion pathways. In addition, the SnO_2_ QDs acts as a “spacer” to prevent the MXene nanosheets from restacking and thus protecting the Li^+^ migration channels and active sites. These unique features endow the 0D–2D SnO_2_ QDs/MXene hybrids with high lithium storage capacity, excellent cycle stability and superior rate performance, indicating a promising anode for LIBs.

## Experimental

### Synthesis of Ti_3_C_2_T_*x*_ MXene

The Ti_3_C_2_T_*x*_ MXene was synthesized by etching Ti_3_AlC_2_ (400 mesh, purchased from Yiyi Technology Co., Ltd.) with LiF + HCl solution as reported previously [41]. Typically, 1.0 g Ti_3_AlC_2_ powder was subjected to a mixture containing LiF (1.0 g) and hydrochloric acid (12 M, 10 mL) under stirring conditions for 24 h at 35 °C. After several times of centrifugation-washing with deionized (DI) water, the product was then dispersed in 50 mL DI water, stored under ultrasound for 30 min. The dark green supernatant was collected by centrifugation at 3500 rpm for 1 h. Finally, the black MXene liquid was obtained and sealed for future use.

### Synthesis of SnO_2_ QDs

In order to obtain SnO_2_ QDs, 4 mmol SnCl_2_·2H_2_O and 4 mmol thiourea (CH_4_N_2_S) were added to 30 mL DI water and magnetically stirred at room temperature to form a milky suspension. After stirring for 24 h, a clear yellow aqueous solution containing SnO_2_ QDs was obtained.

### Synthesis of 0D–2D SnO_2_ QDs/MXene Hybrids

In a typical synthesis, 5 mL of as-prepared SnO_2_ QDs solution was added to 40 mg 0.1 mg mL^−1^ Ti_3_C_2_T_*x*_ MXene solution under ultrasonication for 6 h. The SnO_2_ QDs were deposited on the Ti_3_C_2_T_*x*_ layers during this process and solid–liquid separation was observed. Finally, the black precipitate of SnO_2_ QDs/MXene hybrids were collected by vacuum filtration, washed with water and dried in a vacuum oven at 80 °C for 6 h. The above hybrid was denoted as SnO_2_ QDs/MXene-52. For comparison, we also prepared another SnO_2_ QDs/Ti_3_C_2_T_*x*_ hybrids (SnO_2_ QDs/MXene-51) under the same conditions, but the addition amount of MXene in this solution was 20 mg.

### Materials Characterization

Scanning electron microscopy (SEM) characterization was conducted using a Gemini SEM 500. Transmission electron microscopy (TEM) characterization was conducted on a JEOL JEM-F200 (HR) equipped with selected area electron diffraction (SAED). X-ray diffraction (XRD) patterns were recorded on a Bruker D8 ADVANCE with monochromatic Cu Kα radiation (*λ* = 1.54060 Å). Raman spectra were obtained with a LabRAM HR Evolution Raman spectrometer (633 nm). X-ray photoelectron spectroscopy (XPS) analysis was performed using a Thermo Fisher ESCALAB Xi^+^ to analyze the chemical compositions of the samples.

### Electrochemical Measurements

The working electrodes were prepared by mixing 80 wt% active material, 10 wt% Super P and 10 wt% carboxymethylated cellulose (CMC) in DI water. After coating the slurry on the copper foil, the electrodes were dried at 60 °C in vacuum oven for 8 h to remove the solvent. To test electrochemical performances, CR2025-type coin cells were assembled in an argon-filled glove box (Mikrouna, H_2_O, O_2_ < 0.1 ppm) using Li foil as half-cell counter electrode, and microporous membrane (Celgard 2400) as separator. The electrolyte was 1 M LiPF_6_ in a mixture of ethylene carbonate (EC)/diethyl carbonate (DEC)/dimethyl carbonate (DMC) with a volume ratio of 1:1:1. The charge/discharge (GCD) tests were performed using a LAND BT2000 battery tester. The potential window was 0.01–2.5 V versus Li/Li^+^. The CV traces were recorded on a CS350 electrochemical workstation from 0.01 to 2.5 V. Electrochemical impedance spectroscopy (EIS) measurements were performed on the VSP Bio-Logic SAS at frequencies ranging from 10 mHz to 100 kHz with an applied AC signal amplitude of 10 mV. The capacities of the samples were calculated based on all the components in the active materials, i.e., the mass of SnO_2_ QDs/Ti_3_C_2_T_*x*_ hybrids.

## Results and Discussion

**Fig. 1:**
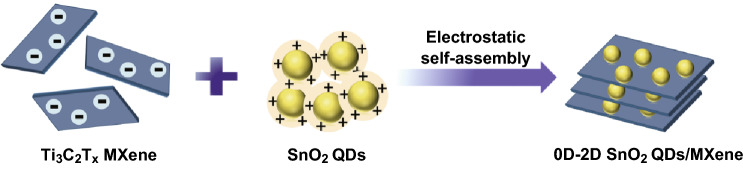
Schematic illustration for the preparation of 0D–2D SnO_2_ QDs/MXene hybrids

The synthesis route for 0D–2D SnO_2_ QDs/MXene by electrostatic self-assembly is illustrated in Fig. 1. Firstly, transparent yellow aqueous solution of positively charged SnO_2_ QDs is prepared by hydrolysis, dehydration and oxidation of SnCl_2_·2H_2_O in DI water, where thiourea is added as a promoter and stabilizer (Fig. S1a). The mercaptan group can easily bound to Sn^2+^, and the SnO_2_ QDs whose zeta potential is +99.0 mV (Fig. S1b) is surrounded by a positively charged protic amino group (–NH_3_^+^), which makes it highly stable and electropositive [42]. The Ti_3_C_2_T_*x*_ MXene solution with its negatively charged groups (–F, –OH) [43], and zeta potential of − 36.2 mV, is very stable in water (Fig. S1a) due to the hydrophilicity and electrostatic repulsion between neighboring nanosheets. When positively charged SnO_2_ QDs are added into the negatively charged Ti_3_C_2_T_*x*_ colloidal solution, the SnO_2_ QDs can easily load onto the surface of the MXene nanosheets. After the continuous ultrasonication of 6 h, the supernatant becomes clear and colorless, and black precipitates are obtained. The positively charged SnO_2_ QDs are captured by the negatively charged MXene nanosheets only by ultrasonic treatment without other components and additional treatments, implying an electrostatic self-assembly mechanism for the designed 0D–2D SnO_2_ QDs/MXene hybrids. The Ti_3_C_2_T_*x*_ MXene with 2D layered structure as a conductive matrix facilitates the charge transfer and accommodates the volume change of SnO_2_ QDs; the SnO_2_ QDs thus prevents the MXene nanosheets from restacking by working as “spacer” and providing channels for fast transfer of Li ion with promising electrochemical performance.

**Fig. 2:**
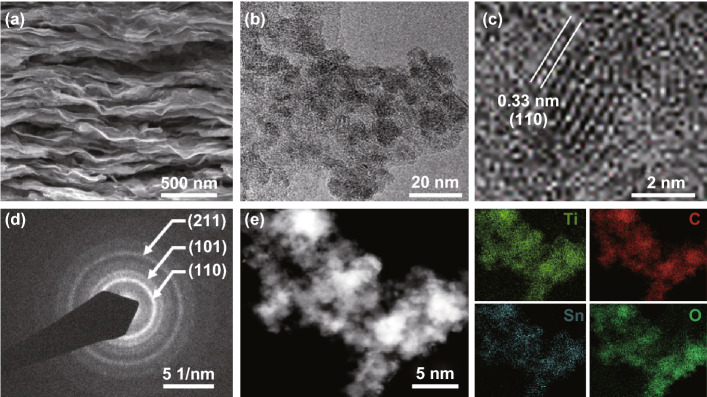
**a** SEM image, **b** TEM image, **c** HRTEM image of 0D–2D SnO_2_ QDs/MXene hybrid. **d** SAED patterns of SnO_2_ QDs. **e** STEM image and corresponding elemental mapping images of Ti, C, Sn, and O

SEM and TEM were employed to characterize the morphology and structure of the as-prepared samples. The pure Ti_3_C_2_T_*x*_ (Fig. S1c) displays a compact 2D layered structure. After decoration with SnO_2_ QDs, the SnO_2_ QDs/MXene hybrids exhibit 0D–2D structure, as shown in Fig. 2a. From a closer view in Fig. 2b, it can be seen that the spatially dispersed SnO_2_ QDs are evenly distributed over the surface of the MXene nanosheets. The particle-size distribution analysis (Fig. S1d) indicates the SnO_2_ QDs have an average particle size of ~ 4.5 nm, favoring the electrode/electrolyte interactions. Since the diffusion of Li ions is strongly dependent on the transport length and the active sites, the ultra-small particle size of SnO_2_ QDs can not only expose a large number of electrochemical active sites shuttling Li ions in and out, but also shorten the diffusion path of Li ion transport, which are beneficial for improving the capacity and rate performance. Figure 2c shows clear lattice fringes of the SnO_2_ QDs, indicating a high degree of crystallinity. The crystal lattice with a spacing of 0.33 nm is consistent with the d spacing of (110) planes of the SnO_2_ tetragonal phase. The selected area electron diffraction (SAED) pattern (Fig. 2d) shows clear diffraction rings, implying the polycrystalline nature of SnO_2_. These diffraction rings can be well indexed to the (110), (101), and (211) planes of SnO_2_. Moreover, the typical scanning TEM (STEM) and elemental mapping images of SnO_2_ QDs/MXene hybrid (Fig. 2e) show homogeneously distributed Ti, C, Sn, and O elements, demonstrating the uniform loading of SnO_2_ QDs on the surface of MXene sheets.

**Fig. 3:**
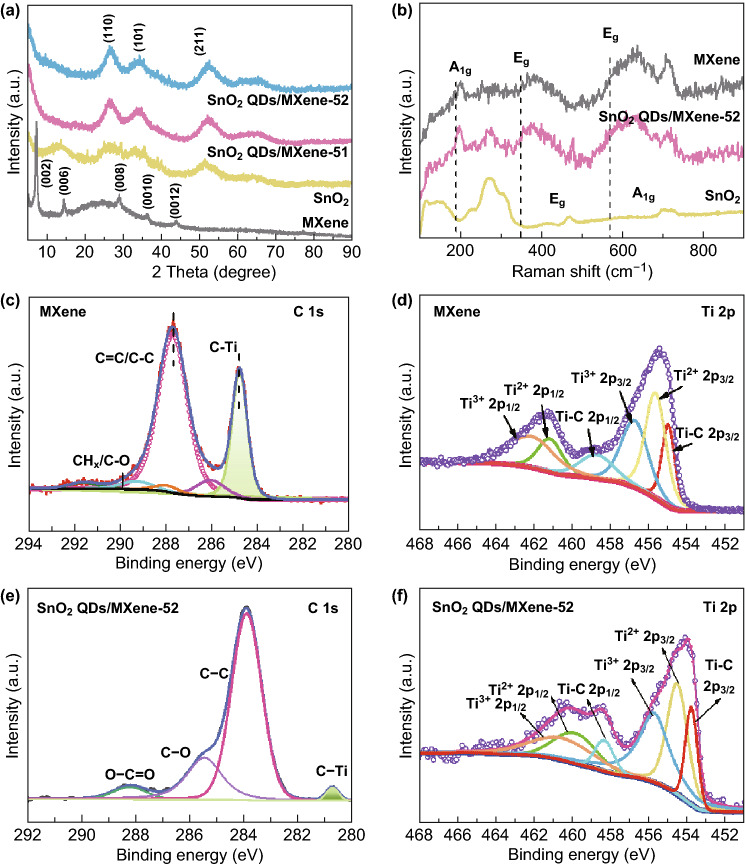
**a** XRD patterns, **b** Raman spectra of MXene, SnO_2_, and SnO_2_ QDs/MXene hybrid. XPS spectra of **c** MXene for high-resolution C 1*s*, **d** Ti *2p*, **e** SnO_2_ QDs/MXene-52 for high-resolution C 1*s*, **f** Ti *2p*

The phase structures of the as-prepared SnO_2_ QDs/MXene hybrids were verified by XRD as shown in Fig. 3a. Ti_3_C_2_T_*x*_ exhibits the major peaks, such as the (002), (006), (008), (0010), and (0012) [44]. Typically, the (002) plane of the Ti_3_C_2_T_*x*_ MXene is located at 2*θ *= 6.4°, corresponding to an interplanar spacing of 13.7 Å [45]. In addition, the pure tetragonal phase of crystalline SnO_2_ (JCPDS No. 41-1445) is located at 26.5°, 34.1°, and 52.4°, corresponding to (110), (101), and (211). The XRD patterns of the SnO_2_ QDs/MXene hybrids consist of Ti_3_C_2_T_*x*_ and tetragonal phase SnO_2_, indicating that SnO_2_ QDs/MXene hybrids have successfully formed. No any extra crystalline phase peaks such as TiO_2_ are observed, indicating the electrostatic self-assembly process is mild with no oxidation of MXene. In addition, the (002) peak of the SnO_2_–MXene composite (Fig. S1e) was downshifted compared to pure MXene, indicating increased interlayer spacing of the MXene in the hybrids.

Raman spectra further confirm the phase characteristics of the SnO_2_ QDs/MXene-52 hybrid. As shown in Figs. 3b and S1f, the peaks of pure SnO_2_ at 271, 465, and 698 cm^−1^ can be assigned to the *E*_g_ and *A*_1g_ active mode of SnO_2_, respectively [46]. Moreover, the pure MXene exhibits a peak at 199 cm^−1^ which corresponds to the *A*_1g_ symmetry out-of-plane vibrations of Ti atoms, whereas the peaks at 381 and 622 cm^−1^ are related to the *E*_g_ group vibrations, including in-plane (shear) modes of Ti, C, and surface functional group atoms [47]. For the SnO_2_ QDs/MXene-52, the spectrum manifests six cognizable Raman-active modes, combining the characteristic Raman peaks of Ti_3_C_2_T_*x*_ and SnO_2_. In comparison with the pure SnO_2_, the peak intensity of SnO_2_ in the hybrid has significantly declined. This suggests that the SnO_2_ QDs are well separated by the Ti_3_C_2_T_*x*_ nanosheets, which weakens the Raman signal from the SnO_2_. It’s worth noting that no strong peak for TiO_2_ was detected at 144 cm^−1^, confirming that MXene has not oxidized during the hybrid formation.

The elemental composition of SnO_2_ QDs/MXene hybrid was further analyzed by XPS. For pristine Ti_3_C_2_T_*x*_ MXene, there are only four main elements, Ti, C, F, and O, whereas for SnO_2_ QDs/MXene-52, the Sn element is also detected, indicating the presence of SnO_2_ in the hybrid (Fig. S2a). In the high-resolution C 1*s* spectrum (Fig. 3c), the characteristic peaks correspond to C–Ti (284.2 eV), C–C (288.7 eV), C–O (288.1 eV), C=O (289.5 eV), and O–C=O (291.6 eV) [48]. The particular peak located at 282.1 eV can be assigned to the C–Ti bond in Ti_3_C_2_T_*x*_ sheets. The XPS spectrum of the Ti 2*p* (Fig. 3d) reveals that peaks of Ti bound to C, Ti(II), and Ti(III). The Ti–C 2*p*_3/2_, Ti(II) 2*p*_3/2_, Ti(III) 2*p*_3/2_, T–C 2*p*_1/2_, Ti(II) 2*p*_1/2_, and Ti(III) 2*p*_1/2_ peaks are detected at binding energies of 455.1, 455.6, 456.8, 458.8, 461.3, and 462.3 eV, respectively. After loading the SnO_2_ QDs, no Ti(IV) peak is observed, suggesting no oxidation of MXene in the SnO_2_ QDs/MXene-52 (Fig. 3f). Meanwhile, the XPS in Sn 3*d* region has two peaks at 487.2 and 495.6 eV, which are attributed to Sn 3*d*_5/2_ and Sn 3*d*_3/2_ of SnO_2_, confirming the formation of SnO_2_ (Fig. S2b). Besides this, the relative intensities of Ti–C, Ti(II), and Ti(III) peaks in SnO_2_ QDs/MXene-52 are relatively weaker than pure Ti_3_C_2_T_*x*_, indicating decreasing Ti_3_C_2_T_*x*_ signal for the hybrid. Furthermore, the slight shift of the Ti–C component to lower binding energy indicates that the synthesis of SnO_2_ QDs/MXene hybrid by electrostatic attraction might have caused shift in the electron density (Fig. 3e). All of these observations demonstrate that SnO_2_ QDs have been successfully deposited on the surface of the Ti_3_C_2_T_*x*_ sheets. In addition, the introduction of SnO_2_ QDs on the Ti_3_C_2_T_*x*_ surface increases the accessible surface area [49], which is evident from the nitrogen adsorption measurements (Fig. S2c). The SnO_2_ QDs/MXene-52 shows a typical type I adsorption/desorption isotherms with a BET specific surface area of 184 m^2^ g^−1^, much higher than that of the pure MXene (19 m^2^ g^−1^). The large surface area is beneficial for accelerating electrolyte diffusion and accommodating volume change of the SnO_2_ QDs during charge/discharge. Thus, much improved lithium storage performances could be expected.

**Fig. 4:**
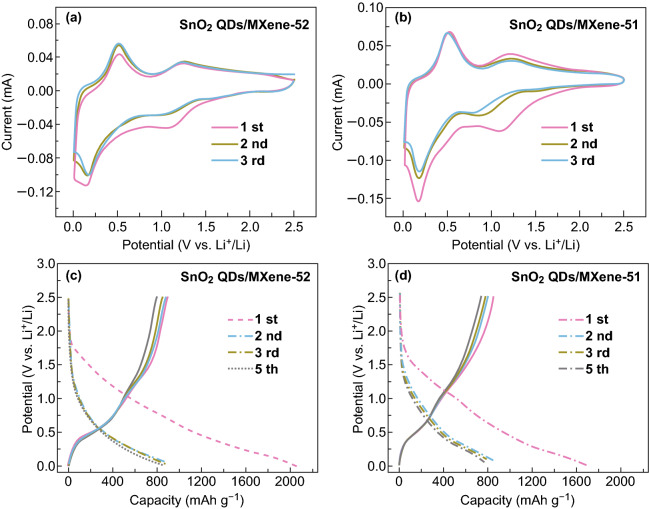
Electrochemical performances as anode in LIBs. **a**, **b** CV curves of SnO_2_ QDs/MXene at a scan rate of 0.1 mV s^−1^ in 0.01–2.5 V. **c**, **d** Charge/discharge curves of SnO_2_ QDs/MXene at 50 mA g^−1^

To evaluate the electrochemical performances of the SnO_2_ QDs/MXene hybrids as anode materials in LIBs, coin-type half cells were assembled using lithium as counter electrode. CV curves of Ti_3_C_2_T_*x*_ MXene and SnO_2_ QDs/MXene hybrids were scanned between 0.01 and 2.5 V. For the pure Ti_3_C_2_T_*x*_ MXene, the broad irreversible reduction peak at around 0.7 V is observed in the first lithiation process, which is attributed to the formation of a solid electrolyte interphase (SEI) generated from the reaction of Ti_3_C_2_T_*x*_ with Li ion [17]. In the subsequent cycles, the reversible peaks near 0.77 and 1.5 V may be the consequence of the reaction between Li^+^ and the titanium-based compounds **(**Fig. S3a) [50]. For the pure SnO_2_ QDs (Fig. S3b), the SnO_2_ is considered to transform into Li_*x*_Sn (*x* ≤ 4.4) and Li_2_O during the initial discharge process (lithium insertion). The cathodic peak at 0.05 V result from the alloying process of Sn to Li_*x*_Sn (0 ≤ *x* ≤ 4.4), and the strong anode peak at about 0.5 V is caused by the de-alloying process of Li_*x*_Sn (0 ≤ *x* ≤ 4.4). The anode peak at 1.2 V is caused by the partial reversible transformation of Sn to SnO_2_ owing to its quantum size [51, 52]. However, in the second and third cycles of the pure SnO_2_, the intensities of the cathodic and anodic peaks show continuous and significant decline, indicating rapid capacity degradation because of the structural variation of SnO_2_ during lithiation/delithiation processes (Fig. S3b). The SnO_2_ QDs/MXene-52 (Fig. 4a) shows CV curves with similar characteristic peaks, but the curves in the 2nd and 3rd cycles almost overlap, indicating good cycle performance. In comparison with the SnO_2_ QDs/MXene-51 (Fig. 4b), the SnO_2_ QDs/MXene-52 display better overlapping CV curves with the same peak positions, implying excellent reversibility in its conversion reaction.

Furthermore, the GCD profiles (Fig. 4c, d) of the SnO_2_ QDs/MXene at 50 mA g^−1^ coincides well with the CV curves. The GCD profile of the Ti_3_C_2_T_*x*_ and SnO_2_ QDs electrodes were also tested at the same conditions (Fig. S3c, d). In the initial discharge profile of the SnO_2_ QDs/MXene hybrids, the plateaus at 0.7 and 0.05 V correspond to the SEI formation and lithium alloying; while during the initial charge, the plateaus at 0.5 and 1.2 V relate to lithium de-alloying and partial reversible SnO_2_ formation, respectively. The reversible capacity of the SnO_2_ QDs/MXene-52 is 887.4 mAh g^−1^ in the first cycle, with initial Coulombic efficiency (CE) of about 51.2%. The low CE might have resulted from the SEI and Li_2_O formation as well as the electrolyte decomposition. In the 5th cycle, the capacity was determined to be about 847.6 mAh g^−1^ and the corresponding CE reached about 100%. With the SEI film protection, the capacity reaches a stable state. In contrast, the SnO_2_ QDs/MXene-51 delivers an initial reversible capacity of 897.5 mAh g^−1^ with a CE of 43.0% (Fig. 4d). The results indicate that the introduction of more SnO_2_ QDs can increase available active sites and the capacity of the hybrid material, but at the same time cause more side reactions and reduce the initial CE, due to its ultra-small particle size and high surface area. Therefore, only appropriate ratio of SnO_2_ QDs and MXene sheets could reach an optimum electrochemical performance.

**Fig. 5:**
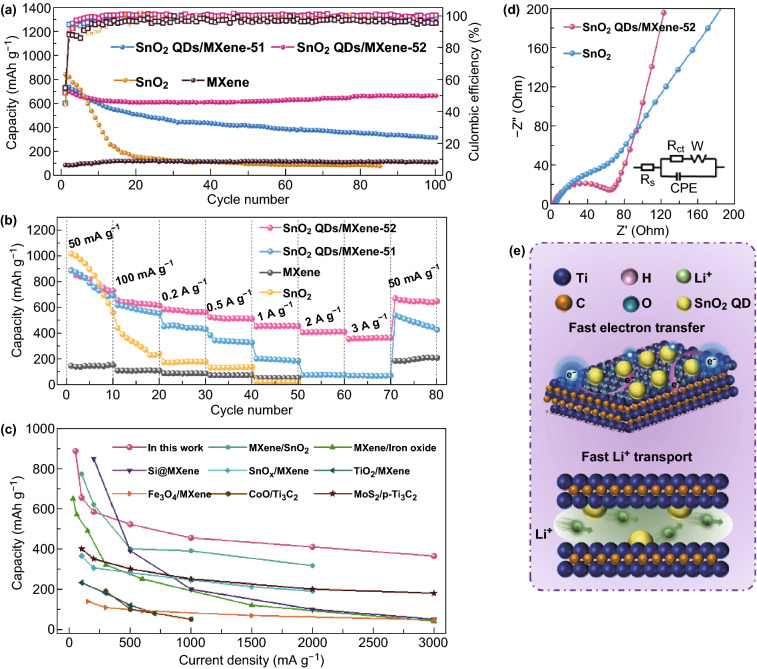
**a** Cycle stability, **b** rate performance of all the samples as LIB electrodes. **c** Comparison of rate capacity between the SnO_2_ QDs/MXene-52 and other MXene-based electrode materials reported for LIBs. **d** Nyquist plots of the SnO_2_ QDs/MXene-52 and the pure SnO_2_ electrodes; **e** energy storage mechanism of the 0D–2D SnO_2_ QDs/MXene hybrids

Figure 5a shows the comparison of the charge/discharge cycle performance among Ti_3_C_2_T_*x*_ MXene, SnO_2_ QDs, and SnO_2_ QDs/MXene at 100 mA g^−1^. The bare Ti_3_C_2_T_*x*_ MXene has a low initial capacity of 79.2 mAh g^−1^ with a CE of 34.2%, which is attributed to the restacking of MXene nanosheets [36]. The initial capacity of the pure SnO_2_ QDs can reach 835.9 mAh g^−1^, but it fades very rapidly due to the severe pulverization of SnO_2_ during charge/discharge. After 20 cycles, the capacity remains only 144.6 mAh g^−1^ with a capacity retention ratio of about 17% of the initial capacity. In contrast, by electrostatic self-assembly of the SnO_2_ QDs on the 2D Ti_3_C_2_T_*x*_ MXene nanosheets, the SnO_2_ QDs/MXene hybrids exhibit much enhanced cycle stability. The capacity of SnO_2_ QDs/MXene-52 reaches to 659.8 mAh g^−1^ with 91% retention of the initial capacity after 100 cycles, which is much higher than that of the pure SnO_2_ QDs and Ti_3_C_2_T_*x*_ MXene. In a comparative evaluation of SnO_2_ QDs/MXene hybrids with different SnO_2_/MXene ratio, it is found that the SnO_2_ QDs/MXene-52 with abundant MXene shows better cycle stability than the SnO_2_ QDs/MXene-51, indicating the importance of adequate MXene substrate in achieving optimum performance [53].

Another attractive feature of the SnO_2_ QDs/MXene electrodes is the excellent rate performance. As shown in Fig. 5b, the SnO_2_ QDs/MXene electrode exhibit much enhanced rate capability compared to pure SnO_2_ QDs. Especially for SnO_2_ QDs/MXene-52, as the current density increases from 50 to 100, 200, 500, 1000, 2000, and 3000 mA g^−1^, its reversible capacity remains 887.4, 655.2, 584.5, 522.0, 454.7, 409.7, and 364.0 mAh g^−1^, respectively, demonstrating superior rate performance. When the current density returns back to 50 mA g^−1^ again, the capacity recovers to 688.1 mAh g^−1^. These results indicate that the unique 0D–2D architecture of the SnO_2_ QDs/MXene hybrids facilitates the Li ion diffusion and the electron transfer, thereby enhancing the reaction kinetics and the rate capability. The comparison of the rate performance for QDs/MXene-52 with previously reported SnO_2_-based anodes and other MXene-based anodes is plotted in Fig. 5c. The SnO_2_ QDs/MXene-52 demonstrate superior rate capability compared to other anode materials based on MXene matrix, such as SnO_2_ QDs/MXene backbone [40], SnO_*x*_ nanosheets/MXene [54], Fe_2_O_3_/Mxene [37], Fe_3_O_4_ nanoparticles@MXene [50], TiO_2_ nanowire/Ti_3_C_2_ [55], CoO nanoparticles/Ti_3_C_2_ [44], MoS_2_/*p*-Ti_3_C_2_ [56], and Si@Ti_3_C_2_ [57] etc.

Electrochemical impedance spectroscopy was employed to compare the reaction kinetics of the SnO_2_ QDs/MXene-52 and the bare SnO_2_ QDs. The typical Nyquist plots of the two electrodes (Fig. 5d) consist of a compressed semicircle in the intermediate frequency region and a diagonal line in the low frequency range. The semicircle is related to the charge transfer resistance (*R*_ct_), and the oblique line is related to Warburg impedance, suggesting the diffusion of Li ion in the active materials [58]. The *R*_ct_ values of the SnO_2_ QDs/MXene-52 and SnO_2_ QDs electrode were calculated to be 75.50 and 100.20 Ω, respectively. Obviously, SnO_2_ QDs/MXene-52 possesses much lower *R*_ct_ value compared to bare SnO_2_ QDs. This can be attributed to the high electrical conductivity of the MXene and the fast charge diffusion reaction due to its unique 0D–2D structure. Moreover, the SnO_2_ QDs/MXene-52 shows a relatively steep low-frequency tail, indicative of high Li ion diffusibility, which results from the efficient ion transfer pathways constructed by MXene.

The above excellent electrochemical performances are because of the synergic effect between MXene nanosheets and SnO_2_ QDs, and the mechanism is illustrated in Fig. 5e. In the SnO_2_ QDs/MXene hybrids, the MXene nanosheets act as 2D substrates for uniform anchoring of SnO_2_ QDs. The MXene nanosheets prevent the aggregation of SnO_2_ QDs and work as an elastic buffer space to adapt to the volume expansion/contraction of SnO_2_ QDs during charging and discharging, thus leading to good cycle stability. Furthermore, MXene nanosheets with good electric conductivity construct effective conductive channels for SnO_2_ QDs, facilitating fast charge transport and improving the rate performance. Moreover, the unique 0D–2D structure offers massive electrochemically active sites for high specific capacity, which contribute in improving the electrochemical performance of electrode materials, thus excellent lithium-ion storage performances are obtained for SnO_2_ QDs/MXene hybrids.

## Conclusions

The 0D–2D SnO_2_ QDs/MXene hybrids have been successfully synthesized by an efficient electrostatic self-assembly strategy. The 0D SnO_2_ QDs with an average size of 4.7 nm are uniformly distributed on the 2D MXene nanosheets with strong adhesion, acting as structurally stable host for lithium storage. The 2D MXene nanosheets buffer the volume change of SnO_2_ QDs during charge/discharge and construct effective channels for charge transport. Besides this, the 0D–2D structure creates additional active sites. The 2D conductive Ti_3_C_2_T_*x*_ MXene, ultra-small SnO_2_ QDs, and unique 0D–2D nanoarchitecture are synergistically responsible for the outstanding electrochemical performances of the SnO_2_ QDs/MXene hybrids. As an anode for LIBs, it exhibits a high specific capacity of 887.4 mAh g^−1^ at 50 mA g^−1^, excellent rate capability (364 mAh g^−1^ at 3 A g^−1^), and superior cycle stability (659.8 mAh g^−1^ after 100 cycles with 91% retention). These results indicate that the 0D–2D SnO_2_ QDs/MXene is a promising anode material for advanced LIBs. In addition, the electrostatic self-assembly method could be extended to other transition metal oxide/MXene hybrids and will have potential applications in sodium-ion batteries, potassium-ion batteries, and supercapacitors.

## Electronic supplementary material

Below is the link to the electronic supplementary material.

Supplementary material 1 (PDF 409 kb)
