# Supplementary material for: Electrostatic Self-assembly of 0D–2D SnO2 Quantum Dots/Ti3C2Tx MXene Hybrids as Anode for Lithium-Ion Batteries
Source: Nanomicro Lett. 2019 Aug 2;11:65. doi: 10.1007/s40820-019-0296-7 (PMC7770891; doi:10.1007/s40820-019-0296-7)
Supplement: Supplementary file 1 — Supplementary material 1 (PDF 409 kb) [file 40820_2019_296_MOESM1_ESM.pdf]

Supporting Information for

Electrostatic Self-Assembly of 0D-2D SnO_2 Quantum Dots/$Ti_3C_2T_x$ MXene Hybrids as Anode for Lithium-Ion Batteries

Huan Liu[1, #], Xin Zhang[2, #], Yifan Zhu[2], Bin Cao[2], Qizhen Zhu[2], Peng Zhang[2], Bin Xu[2, *], Feng Wu[1], Renjie Chen[1, *]

[1]School of Materials Science & Engineering, Beijing Key Laboratory of Environmental Science and Engineering, Beijing Institute of Technology, Beijing 100081, People's Republic of China

[2]State Key Laboratory of Organic-Inorganic Composites, Beijing Key Laboratory of Electrochemical Process and Technology for Materials, Beijing University of Chemical Technology, Beijing 100029, People's Republic of China

[#] Huan Liu and Xin Zhang contributed equally to this work.

*Corresponding authors. E-mail: chenrj@bit.edu.cn (R. Chen); binxumail@163.com (B. Xu)

Supplementary Figures

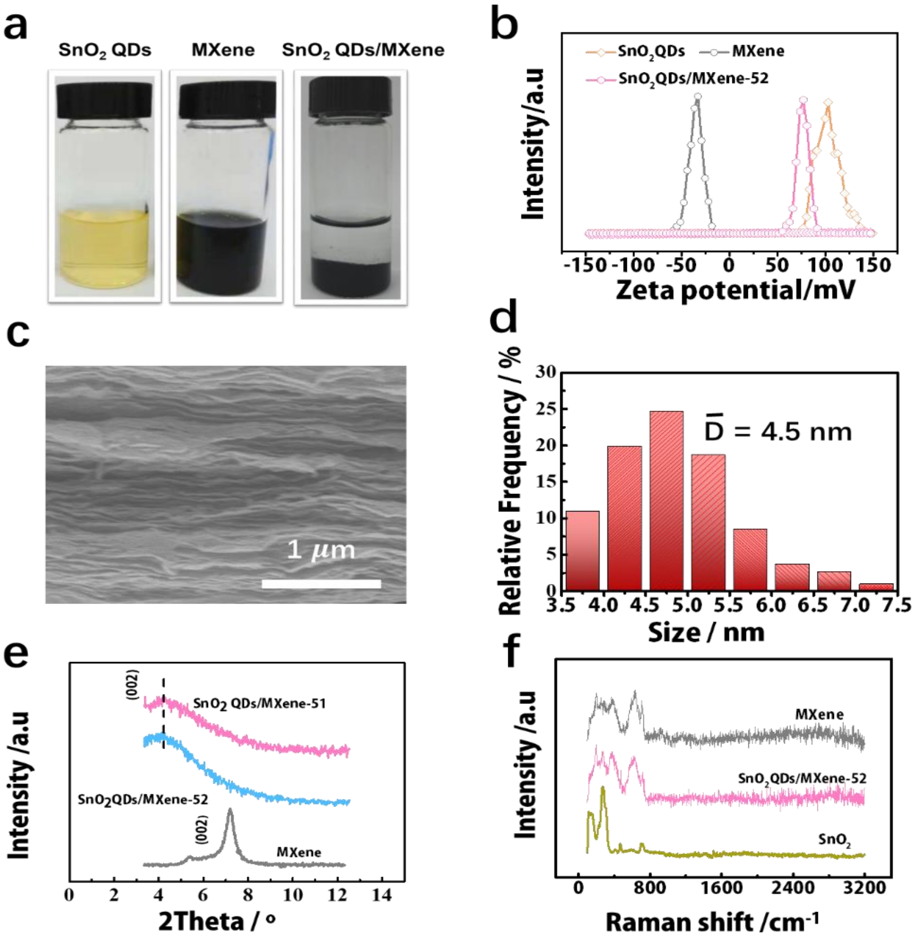

Fig. S1 a Digital photographs of SnO_2 QDs solution, MXene suspension, and SnO_2 QDs/MXene hybrid. **b** Zeta potential of SnO_2 QDs, MXene, and SnO_2 QDs/MXene-2. **c** SEM image of MXene nanosheets, and **d** corresponding particle size distribution of the SnO_2 QDs. **e** XRD patterns in low angle range and **f** Raman spectra of the samples

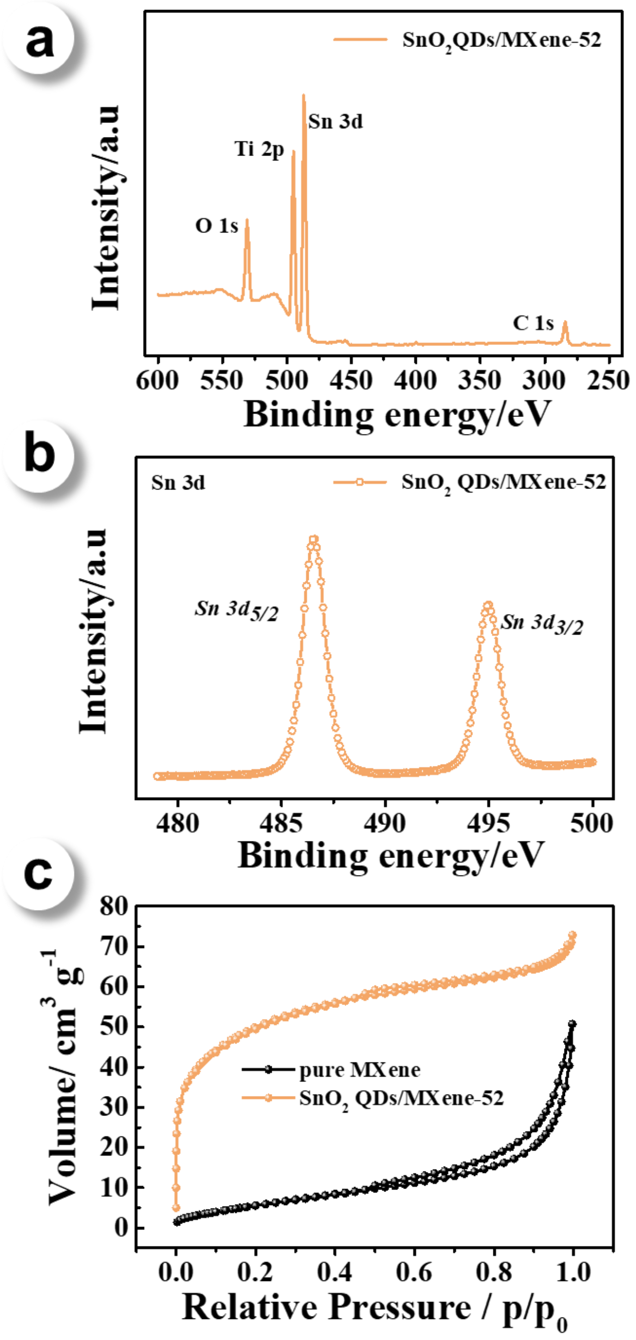

Fig. S2 a XPS curves, **b** the Sn 3d spectrum, and **c** N_2 sorption isotherms of SnO_2 QDs/MXene-52

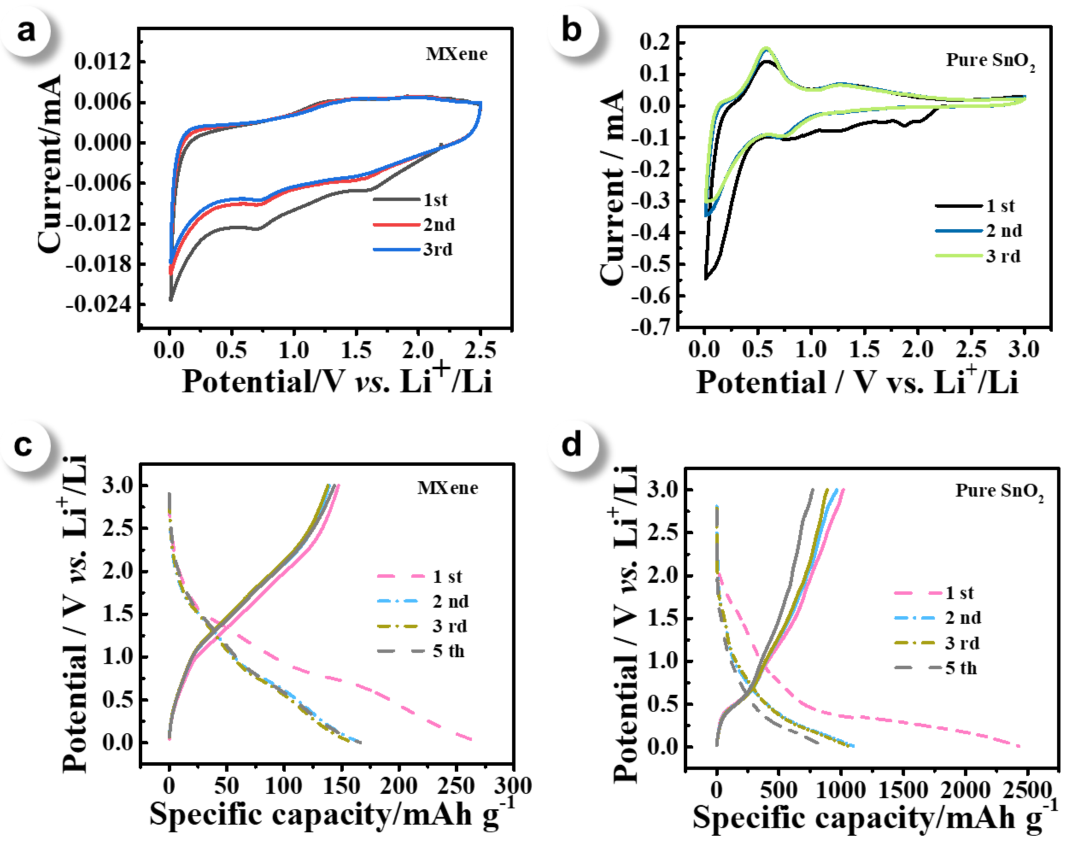

Fig. S3 a CV curves of bare MXene and **b** pure SnO_2 QDs at a scan rate of 0.1 mV s^{-1}; Charge/discharge curves of **c** bare MXene and **d** pure SnO_2 at 50 mA g^{-1}
